# Complete Genome Sequence of *Bacillus paralicheniformis* 14DA11, Exhibiting Resistance to Clindamycin and Erythromycin

**DOI:** 10.1128/genomeA.01216-17

**Published:** 2017-10-26

**Authors:** Jong-Hoon Lee, Do-Won Jeong

**Affiliations:** a Department of Food Science and Biotechnology, Kyonggi University, Suwon, Republic of Korea; b Department of Food and Nutrition, Dongduk Women’s University, Seoul, Republic of Korea

## Abstract

*Bacillus paralicheniformis* 14DA11, exhibiting resistance to clindamycin and erythromycin, was isolated from a Korean fermented soybean food product. The complete genome of strain 14DA11 includes genes that potentially contribute to the antibiotic resistance.

## GENOME ANNOUNCEMENT

The genus *Bacillus* is the most populous bacterial group in Korean fermented soybean foods (1–5). We hypothesized that *Bacillus licheniformis* would be an appropriate doenjang starter candidate among the identified *Bacillus* species, owing to its salt tolerance on tryptic soy agar (Sigma-Aldrich, St. Louis, MO, USA) supplemented with 14% (wt/vol) NaCl (6). Doenjang is a traditional Korean soybean paste, which is ripened at NaCl concentrations of >12% (wt/wt). We previously identified strain 14DA11, which exhibits resistance to clindamycin (MIC, 32 mg/liter) and erythromycin (MIC, 2,048 mg/liter), from our stock cultures in our antibiotic susceptibility test for selecting safe *B. licheniformis* starter candidates for Korean fermented soybean foods (7). The present study involved a complete genome analysis of strain 14DA11 to shed light on the genetic background behind the phenotypic resistance to both antibiotics.

Whole-genome sequencing was performed using the PacBio single-molecule real-time (SMRT) sequencing system by ChunLab, Inc. (Seoul, South Korea). The PacBio reads were assembled using PacBio SMRT Analysis 2.3.0. Gene prediction was performed using CLgenomics (ChunLab), and sequences were annotated by comparison against the Clusters of Orthologous Groups (COG) database (8).

The complete genome of strain 14DA11 consists of a single circular 4,535,069-bp chromosome, with a G+C content of 45.79%. The genome is predicted to contain 4,590 protein-coding sequences, 81 tRNA genes, and 24 rRNA genes. A total of 4,069 genes were functionally assigned to categories based on COG assignments. Gene category analysis shows that the majority of the genes are related to transcription (351 genes [8.6%]), followed by carbohydrate transport and metabolism (346 genes [8.5%]).

Strain 14DA11 has genes coding for type II chitinase, xylosidase, glucanase, and arabinofuranohydrolase. Dunlap et al. separated *Bacillus paralicheniformis* from *B. licheniformis* based on genomic analysis and, in 2015, reported that these four genes are specific to *B. paralicheniformis* (9). Therefore, we conclude that strain 14DA11 is *B. paralicheniformis*.

Furthermore, two potential lincomycin resistance genes, *lmrA* (designated CK945_RS07725) and *lmrB* (CK945_RS05835), and two genes contributing to erythromycin resistance, *ermC* (CK945_RS15790) and *ermD* (CK945_RS19435), were identified. The *ermC* and *ermD* genes are known to endow resistance not only to erythromycin but also to clindamycin (10). Some lincomycin resistance genes have also been reported to be involved in the inactivation of lincosamide antibiotics, including clindamycin (11). Further studies will be required to determine all the orthologs of clindamycin resistance. The complete genome sequence of *B. paralicheniformis* 14DA11 will provide further genetic insight into the strain-specific antibiotic resistance of *Bacillus* species.

### Accession number(s).

The complete genome sequence of *B. paralicheniformis* 14DA11 has been deposited in DDBJ/ENA/GenBank under accession number CP023168.
